# Wine Yeasts Selection: Laboratory Characterization and Protocol Review

**DOI:** 10.3390/microorganisms9112223

**Published:** 2021-10-26

**Authors:** Rossana Sidari, Katarína Ženišová, Blanka Tobolková, Elena Belajová, Tereza Cabicarová, Mária Bučková, Andrea Puškárová, Matej Planý, Tomáš Kuchta, Domenico Pangallo

**Affiliations:** 1 Department of Agraria, Mediterranea University of Reggio Calabria, 89122 Reggio Calabria, Italy; 2 Food Research Institute, National Agricultural and Food Centre, 82475 Bratislava, Slovakia; katarina.zenisova@nppc.sk (K.Ž.); blanka.tobolkova@nppc.sk (B.T.); elena.belajova@nppc.sk (E.B.); tereza.cabicarova@nppc.sk (T.C.); tomas.kuchta@nppc.sk (T.K.); 3 Institute of Molecular Biology, Slovak Academy of Science, 84551 Bratislava, Slovakia; maria.buckova@savba.sk (M.B.); andrea.puskarova@savba.sk (A.P.); matej.plany@savba.sk (M.P.)

**Keywords:** wine yeasts, *Saccharomyces*, non-*Saccharomyces*, enological characteristics, screening, starter selection, micro-fermentations, wine aroma

## Abstract

Wine reflects the specificity of a *terroir*, including the native microbiota. In contrast to the use of *Saccharomyces cerevisiae* commercial starters, a way to maintain wines’ microbial *terroir* identities, guaranteeing at the same time the predictability and reproducibility of the wines, is the selection of autochthonous *Saccharomyces* and non-*Saccharomyces* strains towards optimal enological characteristics for the chosen area of isolation. This field has been explored but there is a lack of a compendium covering the main methods to use. Autochthonous wine yeasts from different areas of Slovakia were identified and tested, in the form of colonies grown either on nutrient agar plates or in grape must micro-fermentations, for technological and qualitative enological characteristics. Based on the combined results, *Saccharomyces cerevisiae* PDA W 10, *Lachancea thermotolerans* 5-1-1 and *Metschnikowia pulcherrima* 125/14 were selected as potential wine starters. This paper, as a mixture of experimental and review contributions, provides a compendium of methods used to select autochthonous wine yeasts. Thanks to the presence of images, this compendium could guide other researchers in screening their own yeast strains for wine production.

## 1. Introduction

Wine reflects the specificity of a *terroir*, including the microbial *terroir* [1]. This is particularly true in spontaneous fermentation by native wine yeasts that nevertheless expose winemakers to well-known complications. Most winemakers prefer to make use of commercial *Saccharomyces cerevisiae* starters, which guarantee predictability and reproducibility of the wines. On the other hand, the extensive use of worldwide-distributed commercial starters leads to organoleptic flattening and uniformization of the wines. Moreover, the positive contribution of non-*Saccharomyces* strains to must fermentation is well established. Non-*Saccharpmyces* species are known to modulate the wine aromatic profile in particular via esterase and β-glucosidase activities, but also to increase the glycerol content, to lower the alcohol content, and to exert proteolytic and pectinolytic activities that lead to enrichment of the aroma profile [2,3,4,5,6,7,8]. Besides *S. cerevisiae* commercial starters, non-*Saccharomyces* commercial starters have become available in recent years [9].

An alternative for preserving the role of the microbial *terroir* is to isolate and select autochthonous *Saccharomyces* and non-*Saccharomyces* strains towards optimal enological characteristics for use as co- or sequential inocula for wine production in the area from which they were isolated.

Enological characteristics to consider in the selection process are divided into technological and qualitative traits [10]. Technological traits (fermentation vigor, ethanol tolerance, resistance to SO_2_, type of growth in liquid media, growth at high and low temperatures) are characteristics useful for efficient fermentation, while qualitative traits refer to those that influence chemical and sensorial composition and properties of wine (acetic acid and sulfuric compound production, production of volatile compounds connected with pleasant notes or off-flavors, enzymatic activities).

The enological characteristics reported above are important for the selection of *Saccharomyces cerevisiae* strains, but they are also useful for studying non-*Saccharomyces* yeasts. It appears crucial to have a complete overview of the traits of yeast strains to select those possessing the best abilities for must fermentation. Moreover, when choosing strains it is necessary to consider the technology of production and the type of product (wines, sparkling wines, botrytized high-sugar wines) where the selected strains will be applied [11,12,13,14,15,16].

Isolation of autochthonous microorganisms in various countries and wine-production regions with the aim of developing region-specific wine starters continues to be attractive [17]. Such research requires sensitive, relevant, and effective scientific methods to assess genetic, biochemical and technological traits characterizing the potential of microorganisms to be used in wine production. In recent years, various new approaches for enological yeast selection have been reported, while others have been optimized or updated [18,19,20,21]. The number of tests used to screen wine strains ranges from a few to many. As examples, colony morphology [22], ethanol resistance [23,24,25,26,27,28], SO_2_ resistance [23,25,26,27,28,29], H_2_S [22,25,26,27,28,29,30,31], enzymatic properties [22,26,27,28,29,32,33], resistance to osmotic stress [24,26,27], acetic acid production and spore formation [31], growth at various temperatures [24], fermentation vigor [31], and gas production [27] have been investigated. Molecular biology approaches were also developed in order to identify and cluster isolated wine yeasts [34,35], and also to screen the yeast communities in wine-related samples through the analysis of the total extracted DNA and RNA [36,37,38]. The latest methods involve very productive scientific tools in genetics and taxonomy but have been found to be unable to replace the more traditional microbiological methods.

To the best of our knowledge, there is a lack of a digest reporting all of the main microbiological methods, including images, to use for selecting wine yeast strains. This paper provides a compendium of up-to-date, complementary, and reliable methods for this purpose, including an outline of an experimentally verified approach for results evaluation and synthesis of conclusions.

The aim of this work was to provide this compendium of methodologies and images to select wine yeasts based on studies of our own isolates. Through assessing the main technological and qualitative enological strain characteristics using media and grape must micro-fermentations, one *Saccharomyces*—*S. cerevisiae* PDA W 10—and two non-*Saccharomyces*—*Lachancea thermotolerans* 5-1-1 and *Metschnikowia pulcherrima* 125/14—strains were selected as potential wine starters.

## 2. Materials and Methods

### 2.1. Yeast Strains

Twenty-nine yeast strains belonging to the Culture Collection of Wine Yeasts (Food Research Institute, National Agricultural and Food Centre, Bratislava, Slovakia) were considered in this study. Twenty-six strains were previously isolated from wine-related samples from various Slovakian regions and three strains—*Candida dubliniensis* CCY 29-178-1, *Metschnikowia pulcherrima* CCY 69-2-15, and *Pichia fermentans* CCY 29-97-12—came from the Culture Collection of Yeasts (Institute of Chemistry, Slovak Academy of Science, Bratislava, Slovakia) (Table 1). The strains were stored in freeze-dried form until the analyses, when they were identified and then tested for technological and qualitative enological characteristics to select *Saccharomyces* and non-*Saccharomyces* strains useful for winemaking. All trials were carried out in duplicate.

### 2.2. Yeast DNA Restriction Analysis and Sequencing

The strains were grown on Yeast Peptone Dextrose (YPD) agar (HiMedia, Mumbai, India) at 25 °C and the biomass was used for molecular analyses. DNA was extracted using InstaGene Matrix (Bio-Rad Laboratories, Hercules, CA, USA) according to the manufacturer’s instructions. The 5.8S Internal Transcribed Spacer (ITS) rRNA region was amplified in a thermal cycler (Bio-Rad, iCycler) using the primers ITS1 (5′-TCC GTA GGT GAA CCT GCG G-3′) and ITS4 (5′-TCC TCC GCT TAT TGA TAT GC-3′) under the following conditions: 25 μL reaction mixture containing 6 μL of DNA template, 1× reaction buffer, 1.2 mM MgCl_2_, 200 μM dNTP mix, 50 pmol of each primer (Microsynth, Balgach, Switzerland), and 1.5 U of DFS-Taq polymerase (Bioron, Ludwigshafen, Germany). The amplification program was: initial denaturation at 94 °C for 2 min, 30 cycles of 10 s at 94 °C for denaturing, annealing for 20 s at 54 °C, extension for 1 min at 72 °C, and a final extension of 8 min at 72 °C.

The ITS-PCR products on 1.5% agarose (Lonza, Basel, Switzerland) gel stained with GelRed stain (Biotium, Fremont, CA, USA) were visualized on a UV transilluminator (UVP Inc., Upland, CA, USA) and then analyzed via Restriction Fragment Length Polymorphism (RFLP) using *Hae*III, *Hha*I (New England Biolabs, Ipswich, MA, USA) and *Hinf*I (Thermo Fisher Scientific, Waltham, MA, USA) restriction enzymes. Restriction mixtures were incubated at 37 °C for 3 h and then analyzed on 2.5% (*w*/*v*) agarose gel at 100 V for 2 h. A representative for each PCR-RFLP profile was chosen for sequence analysis. All of the amplified products were purified using EXO-SAP-IT^®^ (Affymetrix, Cleveland, OH, USA), according to the manufacturer’s instructions. The purified products were prepared according to the instructions of a commercial facility (Eurofins Genomics, Ebersberg, Germany) and shipped to be sequenced via the Sanger method. The sequences were compared with those available at NCBI using Blast search tool [39] and submitted to GenBank (https://submit.ncbi.nlm.nih.gov/subs/genbank/) for accession numbers.

### 2.3. Fourier-Transform Infrared Spectroscopy (FTIR)

The strains were grown on YPD agar at 30 °C for 2-3 days. Subsequently, a loopful from one colony of each strain was dispersed in distilled water, used to load the 96-position ZnSe plate, and dried at 37 °C for 45 min and spectra were measured with a Tensor 27 FTIR spectrometer (Bruker Optics, Ettlingen, Germany) using 32 scans per sample [40].

### 2.4. Yeast Screening

Depending on the test to perform, strains were grown overnight at 25 °C either on YPD agar or in YPD broth and then the cultures were used to inoculate either YPD broth or specific media. For the latter, each strain was harvested by centrifugation (5000 rpm for 10 min), washed once in NaCl 0.9% (*w*/*v*) solution, re-suspended to Optical Density (OD)_600_ of 1.0 in the same solution [41]. Subsequently, each strain was spotted (5 µL) in duplicate onto specific media.

#### 2.4.1. Macroscopic and Microscopic Observation, Type of Growth, CO_2_ Production

Strains were grown in tubes containing YPD broth for 48 h at 25 °C to assess the modality of growth, and in YPD broth tubes with Durham tubes for 7 d at 25 °C to assess fermentation through CO_2_ trapped in the Durham tubes. In addition, the strains were streaked onto YPD agar in order to record the colony morphology. A small quantity of the biomass was observed under a microscope (Olympus BX53, Tokyo, Japan) to record the cell morphology.

#### 2.4.2. Spore Formation

The yeast biomass, taken with a sterile 1 µL loop, was streaked onto sodium acetate (1 g/L) agar (20 g/L) plates to check spore formation after 10 days of incubation at 25 °C [42].

#### 2.4.3. Growth at Various Temperatures

Each strain pre-grown overnight at 25 °C was inoculated at 1% in YPD broth and statically incubated at both 18 °C and 37 °C for 24 h to test its ability to grow at low and high temperatures [24].

#### 2.4.4. Low pH, Ethanol, and Osmotic Tolerance

Each strain culture was spotted onto YPD agar adjusted either to pH 3.0 or supplemented with 300 g/L of glucose or ethanol content (EtOH) of 5%, 10%, 12% or 15%. The plates to test the ethanol tolerance were freshly poured and sealed with Parafilm to prevent evaporation. All inoculated plates were incubated at 30 °C and colony development was checked daily [24].

#### 2.4.5. SO_2_ Resistance

Each strain culture was spotted onto YPD agar adjusted to pH 3.0 and supplemented with various concentrations of potassium metabisulphite [29]. A potassium metabisulphite stock solution was filter-sterilized (pore-size, 0.45 µm) and then added to the medium in concentrations of 80 (only for the strains selected for microvifications), 100, 200, 300, and 400 mg/L in order to correspond to half of the concentrations of SO_2_. The plates were incubated at 30 °C and the colony development was checked daily.

#### 2.4.6. Catalase Activity

The yeast biomass, taken with a sterile 1 µL loop, was added to a drop of 3% (*v*/*v*) H_2_O_2_ [43]. The development of bubbles indicated positive activity.

#### 2.4.7. Acetic Acid Production

A loopful (1 µL) of biomass of each strain was streaked onto Chalk agar (yeast extract 3 g/L, glucose 10 g/L, calcium carbonate 3 g/L, agar 15 g/L) plates and incubated for 7 d at 25 °C [44]. The presence and extent of a clear halo around the yeast biomass indicated the rate of acetic acid production.

#### 2.4.8. H_2_S Production

A loopful (1 µL) of biomass of each strain was streaked onto BiGGY agar plates and incubated for 48 h at 25 °C [45]. The color intensity of the biomass indicated the rate of H_2_S production [20].

#### 2.4.9. β-Glucosidase Activity

Each strain culture was spotted onto Petri plates containing arbutin (5 g/L), yeast extract (10 g/L), 40 drops/100 mL of a 1% solution of ferric ammonium citrate solution, and agar (20 g/L) according to Caridi et al. [46]. After incubation at 25 °C for 7 days, this activity was indicated by the medium changing color, from pale to dark brown.

#### 2.4.10. Pectinase Activity

Each strain culture was spotted onto Petri plates with YNB (6.7 g/L), apple pectin (12.5 g/L), and agar (10 g/L), adjusted to pH 4 with 1 N HCl according to Sidari et al. [47]. After 10 days of incubation at 25 °C, activity was determined by measuring the diameter of the colonies and checking for the presence of a clear halo after flooding the plates with Lugol’s solution and washing with water [27].

#### 2.4.11. Esterase Activity

Each strain culture was spotted onto Petri plates with peptone (10 g/L), NaCl (5 g/L), CaCl_2_·2H_2_O (0.1 g/L), Tween 80 (10 g/L), and agar (20 g/L) at pH 6.8 [48]. After 6 days of incubation at 25 °C, the ability to hydrolyze esters was estimated by the presence of a visible opaque precipitate around the colony.

#### 2.4.12. Protease Activity

Each strain culture was spotted onto Petri plates with a medium prepared by mixing the two following solutions: malt extract 3 g/L, yeast extract 3 g/L, peptone 5 g/L, glucose 10 g/L, NaCl 5 g/L, agar 20 g/L (separately sterilized), adjusted to pH 3.5 with 0.1 M HCl; and a skim milk solution (10% *w*/*v*) prepared and treated at 100 °C for 10 min. After incubation for 3 days at 25 °C, the presence of a clear halo around the yeast spot indicated protease activity [29].

### 2.5. Micro-Fermentation Trials in Grape Must

Eleven out of 29 strains, chosen considering the results of the above-reported screening tests, were tested for fermentation vigor after 2 d and 7 d in Trauben saft—100% Direktsaft red grape must (dmBio; Drogerie Markt, Wals-Siezenheim, Austria), both with and without SO_2_ supplementation. The sugar content of the must was 16 °brix and the pH was 3.20. Aliquots of 100 mL of the must were distributed into flasks, 10 mL of liquid paraffin was added to avoid surface contact with oxygen, and the resulting mixtures were pasteurized at 100 °C for 20 min. Subsequently, half of the flasks were supplemented with 80 mg/L of potassium metabisulphite. All flasks were inoculated in duplicate with 5 mL of 48 h pre-cultures grown in the same red grape must incubated statically at 25 °C. Fermentation progress was monitored by recording weight loss due to the release of CO_2_. After 2 d and 7 d, fermentation vigor was expressed as g of CO_2_/100 mL of must [20].

At the end of the fermentation, the wines produced with and without SO_2_ were analyzed for pH, total titratable acidity (TTA), volatile acidity, ethanol, glucose and fructose, total polyphenols, total flavonoids, and volatile organic compounds (VOCs).

The pH was determined using a pH-meter (OP-208/1, Radelkis, Budapest, Hungary).

The parameters of ethanol, TTA and volatile acidity were determined according to the official methods of International Organisation of Vine and Wine (OIV) [49]. The results were expressed as the mean of four determinations ± standard deviation.

Determination of glycerol and glucose and fructose sugars was accomplished via high performance liquid chromatography (HPLC) using a PU-4003 chromatograph (Pye Unicam, Cambridge, UK) in accordance with the accredited method published by Suhaj and Belajová [50]. Chromatographic separation was performed on the Kromasil 100-5NH2amino column, 250 × 4.6 mm i.d. (EKA Chemicals AB, Sweden), using an RID-10A refractive index detector (Shimadzu, Tokyo, Japan). The RID optical unit was permanently warmed to 40 °C. All wine samples were injected into 20 µL volumes and eluted isocratically with mobile phase acetonitrile:water, 80:20 (*v*/*v*). The flow rate of the mobile phase was 1.5 mL/min. The peaks were identified by retention times and quantified via external calibration using the software QC Expert version 2.5 (TriloByte Statistical Software, Pardubice, Czech Republic). Wine samples were diluted twofold with deionized water and filtered through a 0.45 μm syringe filter with a cellulose membrane (Agilent, Waldbronn, Germany) and subsequently injected for HPLC. During the calibration measurements the correlation coefficients were higher than 0.98 for all analyzed compounds. The refractive index detector responses were linear in the range of 0.4–20 g/L for glycerol and 0.5–50 g/L for glucose and fructose.

Total polyphenols (TPC) and total flavonoids (TFC) were determined using a Shimadzu 3600 UV-VIS-NIR spectrophotometer (Shimadzu, Tokyo, Japan) with an accessory. All experiments were performed in duplicate. A 12% (*v*/*v*) ethanol was used as a reference.

TPC was determined by applying the Folin–Ciocalteu modified method [51]. Briefly, 100 µL of wine sample was appropriately diluted with 12% (*v*/*v*) ethanol, 7.9 mL of distilled water, and 500 µL of Folin–Ciocalteu reagent and mixed in a 20 mL vial. After 10 min, 1.5 mL of 20% sodium carbonate was added, and the contents mixed. Samples were incubated at room temperature in darkness for 60 min, and absorbance was measured at 765 nm. Standard solutions of gallic acid were used to construct the calibration curve (0–1500 mg/L). The results were expressed as gallic acid equivalent (GAE, mg/L).

TFC was evaluated according to the modified method with aluminum chloride [51]. Briefly, 500 µL of wine sample was added to a 10 mL vial containing 1.5 mL of 96% ethanol and 2.8 mL of distilled water. After this, 100 µL of 10% aluminum chloride and 100 µL of 1 M potassium acetate were added, and the contents mixed. After 40 min, the absorbance of the final solution was measured at 415 nm. Standard solutions of quercetin were used to construct the calibration curve. The results were expressed as quercetin equivalent (QE, mg/L).

To analyze VOCs, solid phase micro-extraction (SPME) was carried out using a polydimethylsiloxan-divinylbenzene fiber, coating thickness 65 μm (Supelco, Bellefonte, PA, USA), immersed in 10 mL of wine sample and mixed at 6 Hz on a magnetic stirrer during 30 min at 20 °C. The extracted compounds were analyzed via gas chromatography–mass spectrometry (GC-MS) using a 6890N gas chromatograph (Agilent Technologies, Santa Clara, CA, USA) coupled to a 5973 mass spectrometric detector (Agilent Technologies). The SPME fiber was placed in the inlet of the chromatograph for 2 min at 250 °C so as to desorb the extracted compounds. The gas chromatographic separation took place in a DB-WAXetr high polarity polyethylene glycol column (length 30 m, inner diameter 0.25 mm, stationary phase thickness 0.5 μm; Agilent Technologies) using a temperature program of 35 °C for 1 min, 5 °C for 1 min and 250 °C for 1 min. The split ratio was 10:1. The average velocity of the He carrier gas was 34 cm·s^-1^ at constant flow. An ionization voltage of 70 eV was used. Compounds were identified by comparison of mass spectra with the NIST 14 MS library (National Institute Standards and Technology, Gaithersburg, MD, USA). Analysis was carried out solely at orientation level with relative quantification data expressed as peak area percentage.

### 2.6. Statistical Analysis

Data were analyzed using a one-way ANOVA and Tukey’s test at 5% probability level, using the online tool at https://www.statskingdom.com/doc_anova.html (accesed on 9 March 2021).

## 3. Results

### 3.1. Yeast Strains Grouping and Identification

Table 2 reports the grouping of strains via 5.8-ITS rRNA analysis and RFLP and their molecular identification by sequencing and comparison with the GenBank database.

The ITS amplicons had sizes ranging from 380 to 800 bp. Eighteen RFLP patterns were observed; different profiles were also assigned to strains belonging to the same species (*H. uvarum*, *L. thermotolerans*, *M. pulcherrima*, *P. fermentans*, *T. delbrueckii*, *S. cerevisiae*).

From among the twenty-nine yeast isolates, five were identified as *M. pulcherrima*, five as *T. delbrueckii*, five as *S. cerevisiae*, three as *H. uvarum*, three as *P. fermentans*, two as *L. thermotolerans*, one as *Candida dubliniensis*, one as *Debaryomyces hansenii*, one as *Metschnikowia* aff. *chrysoperlae*, one as *Meyerozyma guilliermondii*, one as *Pichia kluyveri*, and one as *Zygosaccharomyces bailii*.

The accession numbers of the yeast strains sequenced and deposited to GenBank are: MZ207954 *C. dubliniensis* CCY 29-178-1, MZ207959 *D. hansenii* 5-1-6, MZ207966 *H. uvarum* 9-2-1, MZ207967 *H. uvarum* 67/14, MZ207960 *L. thermotolerans* 5-1-1, MZ207958 *L. thermotolerans* 5-1-3, MZ207961 *M.* aff. *crysoperlae* 11-1-4, MZ207962 *M. pulcherrima* 11-1-5, MZ207963 *M. pulcherrima* 11-1-7, MZ207955 *M. pulcherrima* 125/14, MZ207969 M*. guilliermondii* 12-5-1, MZ207968 *P. fermentans* 12-4-4, MZ207970 *P. kluyveri* PDA W 9, MZ207956 *S. cerevisiae* 60/16, MZ207957 *S. cerevisiae* 15-1-552, MZ207953 *T. delbrueckii* 3-16-1, MZ207964 *T. delbrueckii* 21-1-5, MZ207965 *Z. bailii* 24-1-25.

**Figure 1:**
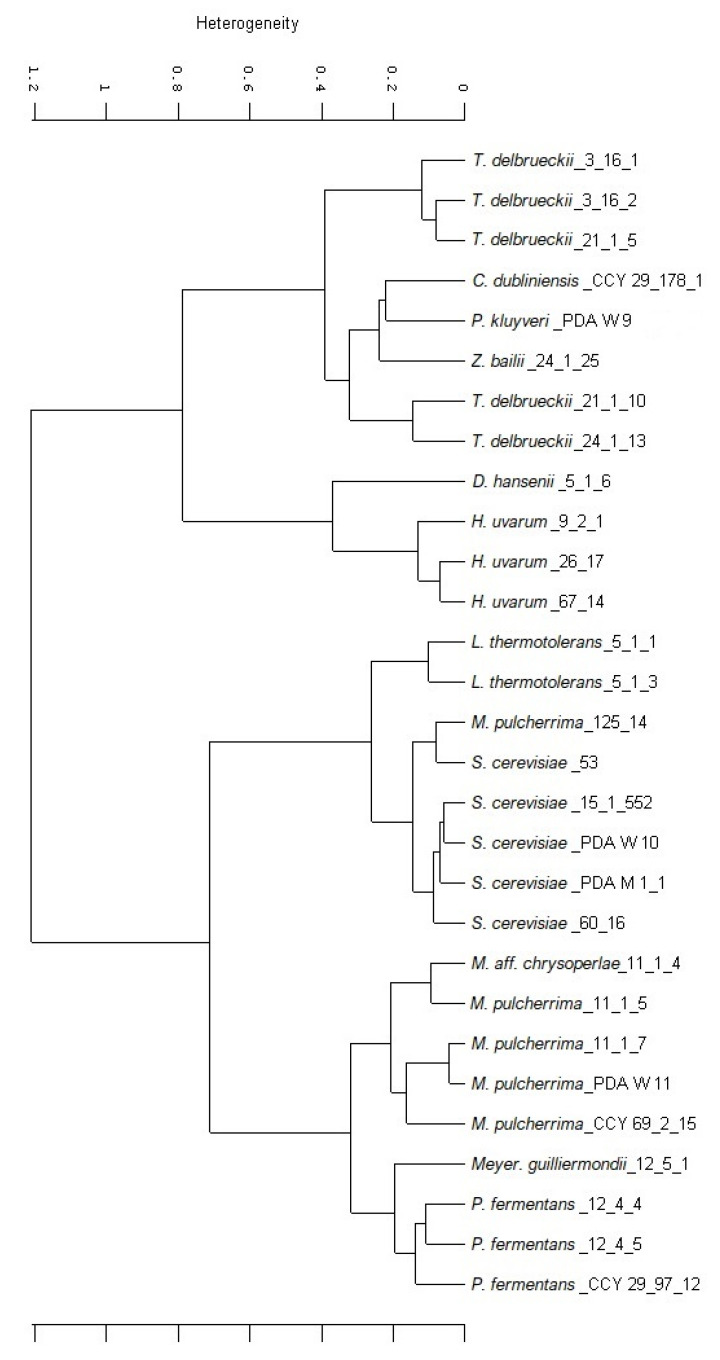
Cluster analysis of the twenty-nine yeast strains as studied by Fourier-Transform Infrared Spectroscopy (FTIR).

Figure 1 reports the clustering of the strains obtained from the FTIR analysis. The mid-infrared range of 4000–500 wavelength/cm^2^ (25,000–2500 nm) is used to excite atoms in molecular bonds, causing them to vibrate. A spectrum can be measured and calculated by light absorption. This specific absorption is then attributed to cell components (e.g., polysaccharides, fatty acids, proteins, mixed region, fingerprint region) [52] used for identification [53]. To enhance the resolution of complex bands and to minimize difficulties evolving from inevitable baseline shifts, the second derivations of the original spectra were calculated. This made it possible to obtain a list of the most similar spectra from the database [54], leading to identification at the species level.

Isolates were classified into sub-clusters by defining a spectral distance as a value for separation on the strain level. The spectral distance chosen was 0.1. The grouping reported in the figure closely corresponds to the results obtained from sequencing.

### 3.2. Yeast Colonies, Cells, and Spores Characteristics

Table 3 reports the morphology, color, and texture of the colonies of the different strains grown on YPD agar. The strains belonging to the *Metshnikowia* genus exhibited biomass turning red during the prolonged incubation time.

**Figure 2:**
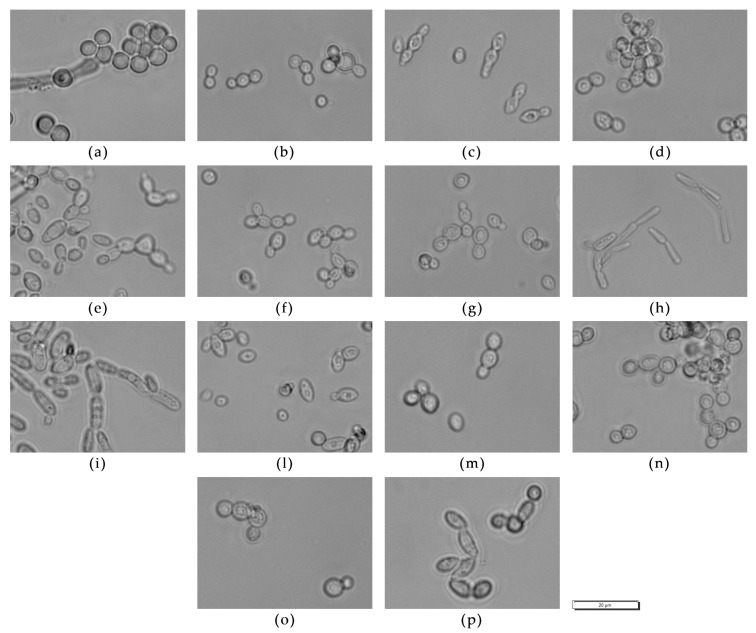
Optical microscopy of representative strains: (**a**) *C. dubliniensis* CCY 29-178-1, (**b**) *D. hansenii* 5-1-6, (**c**) *H. uvarum* 9-2-1, (**d**) *L. thermotolerans* 5-1-3, (**e**) *M.* aff. *chrysoperlae* 11-1-4, (**f**) *M. pulcherrima* 125/14, (**g**) *M. pulcherrima* 11-1-7, (**h**) *M. guilliermondii* 12-5-1, (**i**) *P. fermentans* 12-4-4, (**l**) *P. kluyveri* PDA W 9, (**m**) *S. cerevisiae* 60/16, (**n**) *T. delbrueckii* 21-1-5, (**o**) *T. delbrueckii* 3-16-1, (**p**) *Z. bailii* 24-1-25. Magnification 40×; scale 20 µm.

The microscopic morphologies of strains belonging to the species chosen as representative are reported in Figure 2.

**Figure 3:**
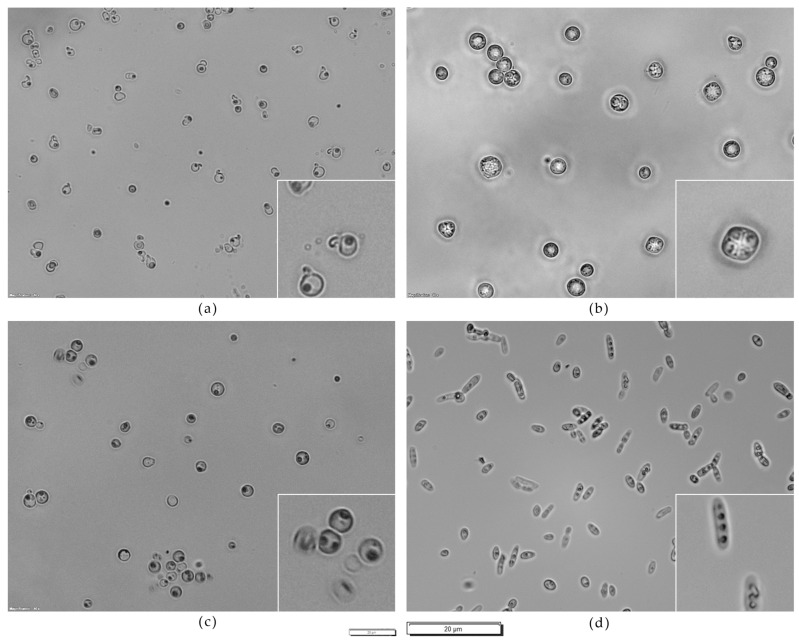
Microscopic observation of (**a**) *D. hansenii* 5-1-6, (**b**) *S. cerevisiae* PDA W 10, (**c**) *T. delbrueckii* 3-16-1, (**d**) *Z. bailii* 24-1-25 grown on sodium acetate agar. Zoomed parts: (**a**) cells with conjugation tubes, (**b**–**d**) spores. Magnification 40×; scale 20 µm (small bar for global views; large bar for detailed views).

*D.hansenii* 5-1-6 exhibited conjugation tubes; *T. delbrueckii* 3-16-1, *Z. bailii* 24-1-25, *S. cerevisiae* PDA W 10, and *H. uvarum* 9-2-1 differentiated spores [55,56,57]. Figure 3 reports on the microscopic observation of selected strains grown on sodium acetate agar.

### 3.3. Yeast Screening

All strains grew in YPD broth as dispersed cells, with the exception of the strain *M. pulcherrima* CCY 69-2-15, which exhibited growth with aggregated cells.

After two days of incubation in YPD broth, the quantity of CO_2_ produced ranged from none (empty Durham tubes) to large (almost-full Durham tubes). The strains *M. guilliermondii* 12-5-1 and *D. hansenii* 5-1-6 did not produce gas, while the strain *C. dublinensis* CCY 29-178-1 produced a small quantity. The strains belonging to the *M. pulcherrima* species (strains 11-1-7, 125/14 and CCY 69-2-15) and the strains *T. delbrueckii* 3-16-1 and 3-16-2, *S. cerevisiae* 60/16, 15-1-552, 53, PDA W 10, PDA M 1/1, *L. thermotolerans* 5-1-3 and 5-1-1, *M.* aff. *chrysoperlae* 11-1-4 showed moderate gas production (half-full Durham tubes), the strains *M. pulcherrima* 11-1-5, *T. delbrueckii* 21-1-5, 21-1-10, 24-1-13, *Z. bailii* 24-1-25, *M. pulcherrima* PDA W 11, *H. uvarum* 9-2-1 and 67/14, *P. fermentans* 12-4-5 and CCY 29-97-12 were characterized by above-average gas production (more than half-full Durham tubes), and the strains *H. uvarum* 26/17, *P. fermentans* 12-4-4, and *P. kluyveri* PDA W 9 exhibited large gas production (almost full Durham tubes). After three or more days, all strains showed a very large CO_2_ production (full Durham tubes) except the strains *M. guilliermondii* 12-5-1 and *D. hansenii* 5-1-6, which did not ferment. Moreover, after tube vortexing, the strains *H. uvarum* 67/14 and 26/17, *P. fermentans* 12-4-4 and 12-4-5, and *P. kluyveri* PDA W 9 produced thick and persistent foam.

Five out of twenty-nine strains exhibited veils: *M. pulcherrima* 125/14 (weak), *P. fermentans* 12-4-4 and 12-4-5, *P. kluyveri* PDA W 9 (abundant), *P. fermentans* CCY 29-97-12 (abundant and thick).

Concerning growth at 37 °C, the strains *T. delbrueckii* 3-16-1 and 3-16-2, *D. hansenii* 5-1-6, *L. thermotolerans* 5-1-1, *M.* aff. *chrysoperlae* 11-1-4, *M. pulcherrima* 11-1-5 and CCY 69-2-15, *T. delbrueckii* 21-1-5, 21-1-10, 24-1-13, *Z. bailii* 24-1-25, and *H. uvarum* 9-2-1, 26/17 and 67/14 were unable to grow. Strains showing moderate to intense growth were, in order: *S. cerevisiae* 53, *C. dubliniensis* CCY 29-178-1, *M. pulcherrima* 125/14, *S. cerevisiae* 60/16, 15-1-552, PDA W 10, and PDA M 1/1, *P. fermentans* 12-4-5, and PDA W 9 *P. kluyveri*. Strains that exhibited growth as poor pellets were *M. pulcherrima* PDA W 11 and *P. fermentans* 12-4-4, while the strains *L. thermotolerans* 5-1-3, *M. pulcherrima* 11-1-7, and *M. guilliermondii* 12-5-1 showed some clouding of the culture broth.

Concerning growth at 18 °C, all strains were able to grow to a certain degree; ordered from lowest to most intense growth: *M.* aff. *chrysoperlae* 11-1-4, *S. cerevisiae* 53, *M. pulcherrima* 125/14, *S. cerevisiae* PDA W 10, PDA M 1/1, *L. thermotolerans* 5-1-3, 5-1-1, *H. uvarum* 9-2-1, 67/14, 26/17, *P. fermentans* 12-4-4, 12-4-5, CCY 29-97-12, *T. delbrueckii* 3-16-1, 3-16-2, *C. dubliniensis* CCY 29-178-1, *S. cerevisiae* 60/16, 15-1-552, *M. pulcherrima* 11-1-5, 11-1-7, CCY 69-2-15 *M. pulcherrima*, *T. delbrueckii* 21-1-5, 21-1-10, 24-1-13, *M. pulcherrima* PDA W 11, *P. kluyveri* PDA W 9, *D. hansenii* 5-1-6, *Z. bailii* 24-1-25, and *M. guilliermondii* 12-5-1.

The ability of the strains to grow under the stressed conditions possibly occurring during must fermentation was studied to consider their potential application in vinification. All strains were able to grow well in an osmotic stress condition (300 g/L of glucose) and at pH 3.0. Table 4 reports the biochemical activities of the twenty-nine strains tested.

Concerning ethanol tolerance after one day of incubation (Table 4a), all strains grew well in the medium supplemented with 5% of ethanol, while differences were observed with increasing ethanol concentration. In particular, with 10% of ethanol three strains grew very well—*S. cerevisiae* PDA M 1/1, *L. thermotolerans* 5-1-3, *P. kluyveri* PDA W 9. The strains that tolerated 12% of ethanol to various extents were those belonging to *S. cerevisiae*, *L. thermotolerans*, *M. pulcherrima* 125/14, *P. kluyveri* PDA W 9, *Z. bailii* 24-1-25, and two strains of *T. delbrueckii*. This trend was observed also in the presence of 15% of ethanol, with the exception of the strains *P. kluyveri* PDA W9 and *Z. bailii* 24-1-25, which did not grow.

After one day of incubation in media supplemented with increasing concentrations of SO_2_ (Table 4a), *S. cerevisiae* strains showed good growth indicating full resistance (to 400 mg/L of metabisulphite), with the exception of the *S. cerevisiae* 60/16 which did not experience good growth with more than 200 mg/L of metabisulphite. This strain did show good growth in media supplemented with 300 and 400 mg/L of metabisulphite after two and three days of incubation, respectively. Non-*Saccharomyces* strains showed marked differences in a strain-dependent manner. The strains belonging to *Lachancea*, *Debaryomyces*, *Hanseniaspora*, and *Meyerozyma* genera exhibited no growth even at the lowest SO_2_ concentration. Some of the strains needed longer incubations to confirm their incapability to grow in presence of SO_2_ or to resist at different concentrations. As example, *L. thermotolerans* 5-1-1 grew after two days of incubation in the presence of 100 mg/L of metabisulphite, while at the highest SO_2_ concentration only *P. kluyveri* PDA W 9 and the above mentioned *S. cerevisiae* 60/16 improved their growth over the incubation period.

All eleven strains chosen for the microvinification trials exhibited good growth in the presence of 80 mg/L of metabisulphite.

All strains were catalase-positive, with non-*Saccharomyces* strains exhibiting the highest activity, especially strains belonging to the species *T. delbrueckii*, *D. hansenii*, *M. pulcherrima*, *H. uvarum*, and *P. fermentans* (Table 4b).

**Figure 4:**
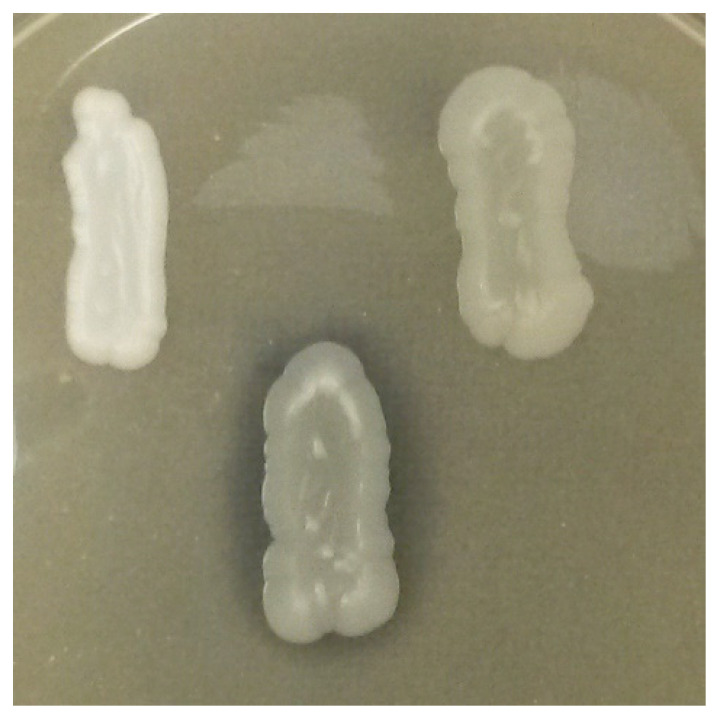
Yeast strains’ acetic acid production according to the absence/presence of a halo around the biomass grew on 90 mm Petri plate. Clockwise from left: negative strain *M. pulcherrima* PDA W 11, positive strains *H. uvarum* 9-2-1 and *H. uvarum* 67/14 exhibiting different degrees of production.

Only 6.9% of the strains were positive for acetic acid production. These belonged to the species *H. uvarum* (Figure 4).

**Figure 5:**
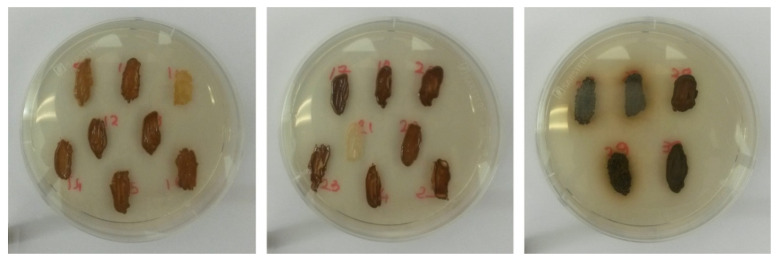
Different degrees of H_2_S production among yeast strains according to the color of the biomass; white: low production, hazel: medium production, dark: high production.

The strains exhibited a wide range of H_2_S production with the biomass color ranging from white to black and passing through intermediate tints (Figure 5). The majority of the strains (41.38%) had hazel biomass followed by dark hazel (37.93%), black (13.79%), pale hazel (3.45%), and white (3.45%) (Table 4a).

**Figure 6:**
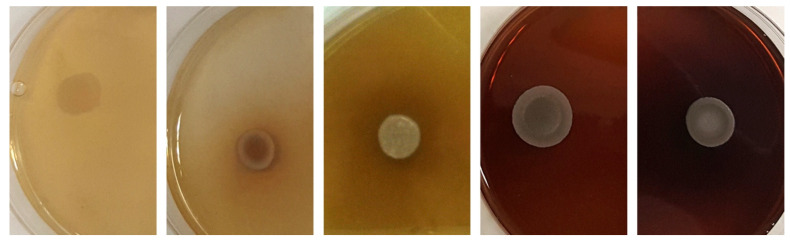
Different degrees of β-glucosidase activity among yeast strains according to the absence/presence of brown color in the medium. From left to right: *S. cerevisiae* 60/16, *D. hansenii* 5-1-6, *M. pulchrerrima* 11-1-5, *M. pulcherrima* 125/14, *M. pulcherrima* 11-1-7.

Thirty-one percent of the strains exhibited light to strong β-glucosidase activity (Figure 6). The highest activity was recorded for strains belonging to the genus *Metschnikowia* and the strain *P. kluyveri* PDA W 9 (Table 4b).

**Figure 7:**
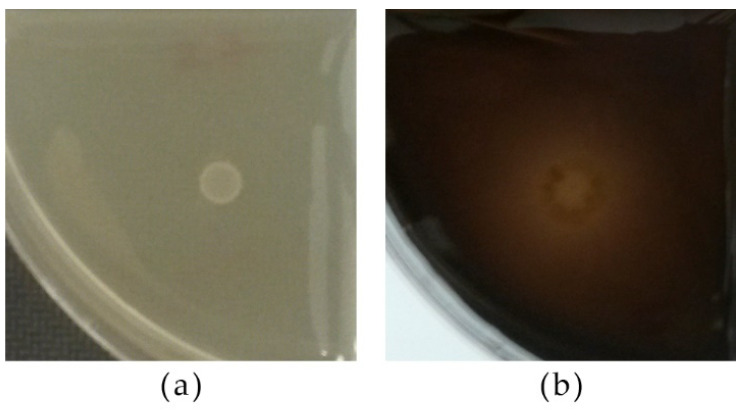
Pectinase activity of yeast strains according to growth on the medium and the presence of a clear halo after washing with Lugol’s solution; (**a**) *P. fermentans* 12-4-5, (**b**) *P. kluyveri* PDA W 9.

All strains grew on the medium supplemented with pectin. The strains *C. dublinensis* CCY 29-178-1, *P. fermentans* 12-4-4 and CCY 29-97-12 exhibited wider biomass spots. Moreover, 13 out of 29 strains exhibited a halo around the biomass after flooding with Lugol’s solution (Table 4b). Shortly after washing, the strongest halos remained visible while the faint ones rapidly disappeared (Figure 7).

**Figure 8:**
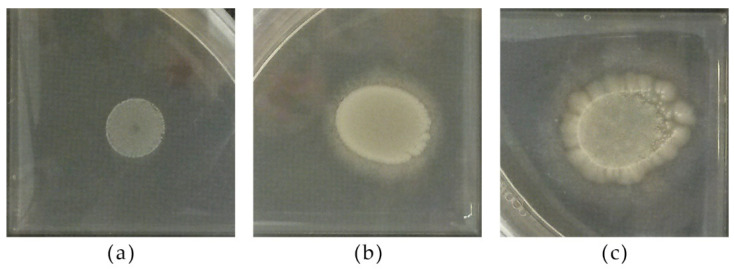
Different degrees of esterase activity among yeast strains according to the presence of precipitate around the biomass: (**a**) no activity (*S. cerevisiae* 53); presence of activity in (**b**) *M. pulchrerrima* 11-1-5 and (**c**) *C. dubliniensis* CCY 29-178-1.

The esterase activity of strains ranged from absent to strong (Figure 8). Only *C. dubliniensis* CCY 29-178-1, *M.* aff. *chrysoperlae* 11-1-4 and *M. pulcherrima* 11-1-5 exhibited high esterase activity while two strains—*M. pulcherrima* 125/14 and *M. guilliermondii* 12-5-1—were characterized by moderate activity (Table 4b).

**Figure 9:**
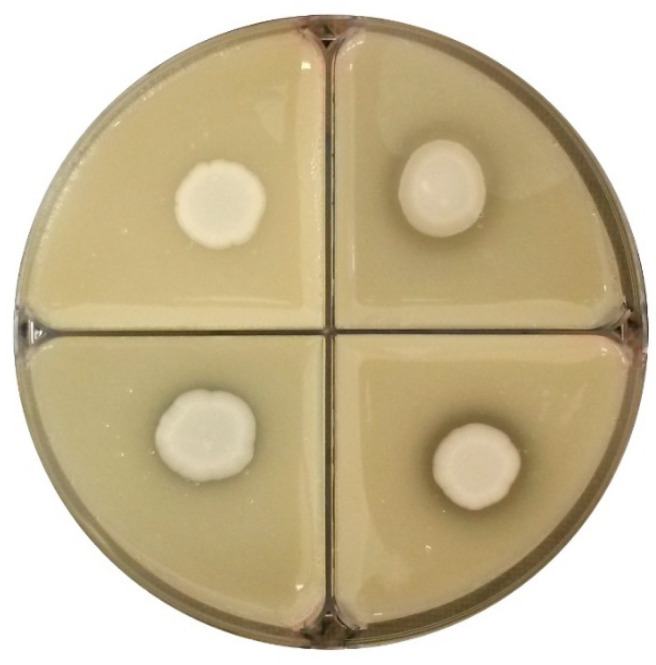
Yeast strains’ protease activity according to the absence/presence of a halo around the biomass. Clockwise from left: negative strain *T.* *delbrueckii* 21-1-5, strains with different degrees of activity *M.* aff. *chrysoperlae* 11-1-4, *S. cerevisiae* 60/16, *T. delbrueckii* 21-1-10.

A total of 58.62% of strains were positive for protease activity. Those with the highest activity were among the non-*Saccharomyces* yeasts (*Metschnikowia*, *Hanseniaspora*, and *Pichia*) (Table 4b; Figure 9).

Eleven of the twenty-nine strains—*D. hansenii* 5-1-6, *H. uvarum* 26/17, *L. thermotolerans* 5-1-1, *M. pulcherrima* 125/14, *M. pulcherrima* 11-1-7, *S. cerevisiae* 15-1-552, *S. cerevisiae* 53, *S. cerevisiae* PDA W 10, *S. cerevisiae* PDA M 1/1, *T. delbrueckii* 3-16-1, *Z. bailii* 24-1-25—were chosen for micro-fermentation trials considering the results of the screening tests. The choice was made balancing the screening parameters’ results for each strain while also taking into account the possibility of further improving their characteristics through different techniques, such as hybridization. In detail, the strains chosen exhibited no or very low acetic acid production, low to medium H_2_S production, good ethanol and SO_2_ tolerance after one or two days, and a varied range of catalase, β-glucosidase, esterase, pectinase, and protease activities (Table 4).

### 3.4. Enological Characterization by Micro-Fermentations

The eleven strains selected through the screening process were tested using micro-fermentations in order to evaluate their fermentation performance. The non-*Saccharomyces* strains showed lower fermentation vigor than the *S. cerevisiae* strains, which exhibited the highest fermentation vigor. Similar observations were reported for *M. pulcherrima* 125/14. The lowest values reported were for *D. hansenii* 5-1-6. Similar results were reported for fermentation vigor without and with SO_2_, confirming the results obtained by screening on plates and indicating the resistance of the strains to the used SO_2_ concentration (Table 5).

Table 6, Table 7 and Table 8 show the physicochemical parameters of the wines produced using the eleven selected yeast strains. pH, TTA, and volatile acidity values for the wines produced with each strain are reported in Table 6. All produced wines had a pH higher than the un-inoculated musts, and the TTA values were linearly correlated to pH. The pH range for the trials without SO_2_ was 3.34–3.48, while for the trials with SO_2_ it was 3.29–3.44. The volatile acidity ranged from 0.12 to 1.68 g/L of acetic acid in the absence of SO_2_.

Concerning ethanol production (Table 7), *S. cerevisiae* strains consumed glucose and fructose as expected, with produced ethanol percentages as high as 8.39% and 8.23% by *S. cerevisiae* 15-1-552 without SO_2_ and *S. cerevisiae* PDA W10 with SO_2_, respectively. *M. pulcherrima* 11-1-7 fermented 33 and 30 g/L of glucose in micro-fermentation without and with SO_2_, respectively, and 23 and 19 g/L of fructose in micro-fermentation without and with SO_2_, respectively, producing the lowest percentages of ethanol (4.15% and 4.10% without and with SO_2_). *L. thermotolerans* 5-1-1 fermented 80 g/L of glucose in trials without SO_2_ and consumed almost all of the glucose in trials with SO_2_; it fermented 70 and 81 g/L of fructose in the absence and presence of SO_2_, respectively, producing the highest values of ethanol (approximately 7.15–7.25%). Moreover, *T. delbrueckii* 3-16-1 produced wines with approximately 7% of ethanol. The lowest ethanol production was recorded for *D. hansenii* 5-1-6.

Among the non-*Saccharomyces* yeasts, *M. pulcherrima* 11-1-7 produced wines with the highest glycerol concentration, followed by *Z. bailii* 24-1-25, while the lowest concentration was produced by *D. hansenii* 5-1-6 (Table 8).

Comparing the total concentrations of polyphenols and flavonoids between the inoculated and un-inoculated musts (Table 8), in wines without SO_2_, increases or decreases in concentrations of polyphenol and flavonoid were reported in a strain-dependent manner. By contrast, in wines produced with SO_2_, a decrease in concentration of flavonoids was observed for all strains tested (Table 8).

During GC-MS analysis, various volatile aroma compounds were detected. Most of them are well known as contributors to wine aroma [58,59]. All fermented samples, with the exception of that fermented by *D. hansenii* 5-1-6, contained high amounts of 2-phenylethanol, which is an established aroma compound with a sweet, floral, rosy character. The highest amounts of this compound were produced, among samples fermented with SO_2_ (Table S1), by *L. thermotolerans* 5-1-1. Only samples fermented by *S. cerevisiae* strains, together with that fermented by *M. pulcherrima* 125/14, contained remarkable amounts of 4-vinylguaiacol, which is an aroma compound with a sweet-smoky character, typical for Traminer wines or for whisky. Various strains produced medium-chain fatty acids, a phenomenon more pronounced in samples with SO_2_ (Table S1). Several samples contained considerable amounts of pyran and furan derivatives, which were probably sourced or metabolized from the UHT-treated substrate. Dodecanoic acid was detected only in various fermented samples treated with SO_2_, while only some fermented samples without SO_2_ (Table S2) contained propylene glycol, 4-cyclopentene-1,3-dione, diethyleneglycol ethylether, 2-methylthiolane, hexanoic acid and nonanoic acid.

## 4. Discussion

This contribution aimed to give a guide as comprehensive as possible to methods for wine yeast selection while studying our own strains. We decided to test the strains for all the characteristics to obtain for each of them a complete profile in view of possible genetic improvement. The simple trials used allowed us to exclude those strains possessing the worst features (alone or in combination) for wine-making—high acetic acid and H_2_S production, low ethanol and SO_2_ tolerance, foam production, zero or low enzymatic activity—selecting the best strains to test in must fermentations.

The strains here reported as *M. pulcherrima* are to be considered *M. pulcherrima*-like strains due to the difficulty in assigning an exact taxonomic position, as a result of a lack of distinctive morphological and physiological properties among species belonging to the *M. pulcherrima* clade and the lack of rDNA barcode gaps [60,61,62,63]. In contrast to Sipiczki [63], our strain of *M.* aff. *chrysoperlae* is a pigmented strain, as are all of our strains of *M. pulcherrima*. In our study, since it is known that for taxonomical proposes it is necessary to use more gene markers in order to well classify yeast strains, we chose to use only ITS fragment sequencing as an identification tool in combination with the RFLP and FTIR approaches. The sequencing of ITS regions is suitable as a rapid and preliminary identification tool for yeasts, which can then be deeply taxonomically analyzed exploiting other molecular markers as shown by previous studies [60,61,62,63].

FTIR spectroscopy facilitates the grouping of yeasts based on the chemical composition of their cells. It is a high-throughput method requiring no chemicals to be used, and therefore is cheap and convenient. Our results presented in Figure 1 demonstrate the overall success of this method to group yeast strains similarly to the sequencing-based approach, which is much more tedious and costly. Based on this, and based on our experience and several other studies [40,53,54], we can recommend the use of FTIR spectroscopy for preliminary grouping of strains and reducing the number of strains in order to pass to further evaluation, by elimination of those that are most probably duplicates or multiplicates.

The initial yeast concentration used in the screening might differ among yeast strains and species due to different morphology and size; therefore, we compared each strain with its own control condition, avoiding the comparison of different yeast species.

The presence of sulfur off-flavor in wine as a result of yeast metabolism is negatively correlated to wine quality, as it is an undesired wine off-flavor, and it also gives rise to health concerns. The screening of yeast strains that produce zero or low H_2_S is particularly necessary to take into account for the production of organic and sulfite-free wines. The degree of H_2_S production by non-*Saccharomyces* yeasts and the wide intra-species variability observed by different authors are consistent with the findings of this study [28,29,64]. By contrast, our findings for *H. uvarum* and *M. pulcherrima* conflict with the results of Polizzotto et al. [22] and Belda et al. [65] who reported absent or low sulfite-reductase activity.

The ability of the non-*Saccharomyces* strains under study to grow under stressed conditions was assayed to understand their potential application in vinification. The strains’ ability to grow at low pH and in high concentrations of glucose makes them suitable for harsh environments; in addition, their growth at different temperatures makes them suitable for red and white vinification. Our results confirm that *S. cerevisiae* is the most ethanol-tolerant species compared to many non-*Saccharomyces*. Concerning the non-*Saccharomyces* strains, some of them exhibited higher ethanol tolerance (up to 12% or to a lesser extent up to 15%) compared to results from the literature [28,62,63,64,66,67,68], confirming the results of Mukherjee et al. [69] for *L. thermotolerans*, *T. delbrueckii* and *Z. bailii*. The use of SO_2_ in winemaking is mandatory to control spoilage and microorganisms and to protect wines from oxidation. Therefore, it is important for wine yeasts to be able to tolerate SO_2_ at the dosage commonly used for commercial wine fermentation; on the other hand, the health aspect has to be taken into account. For this reason, although all strains were tested at increasing concentrations of SO_2_ (100–400 mg/L), the microvinification trials were carried out using a low SO_2_ concentration (80 mg/L). Yeasts belonging to *Torulaspora*, *Metschnikowia*, *Zygosaccharomyces*, and *Lachancea* genera were less sensitive to SO_2_ than commonly considered [23] and this is consistent with other authors’ results [28,29]. Concerning the strains’ contribution to the pH of wine, our strain of *L. thermotolerans* confirms the existing strains’ variability in producing lactic acid and consuming malic acid [66]. The selected *L. thermotolerans* strain did not significantly influence pH and total acidity compared to the *S. cerevisiae* controls (53, PDA W 10, PDA M1/1) (Table 6). Previous studies report variability in lactic acid production from 0.2 g/L to approximately 10 g/L and reductions in pH from insignificant differences to 0.5 [70,71,72]. However, many other quality parameters can be improved by *L. thermotolerans*, so the choice of strain can be interesting. Results from the catalase test gave information on the ability of strains to cope with oxidative stress and to perform better during fermentation [73]. All of the strains tested in this study were catalase positive to various extents and in agreement with other authors for *H. uvarum*, *Candida*, *Pichia*, *D.* *hansenii*, *M. pulcherrima* [27,28].

The wine industry makes use of protease and pectinase to prevent wine haze and facilitate wine clarification, together with glycosidase to favor the expression of grape varietal aromas [74]. Wine yeasts can possess one or more natural enzymatic activities useful for vinification [32,61,75,76,77]. Yeast enzymes of interest include esterases, glycosidases, proteases, and cellulases able to hydrolyze structural components [78,79] that determine, based on their presence and intensity, the sensorial complexity of wines [80].

Our results regarding β-glucosidase, which breaks down glycosidic complexes releasing terpenes and other volatile compounds, in our selected yeast strains are consistent with other authors reporting high β-glucosidase incidence in *Debaryomyces* and *Pichia* [65,77,81] and especially in *M. pulcherrima*, mostly in a strain-dependent manner [28,29,32,65,77,81,82,83]. Our strains positive for esterase, which hydrolyzes long-chain esters, belonged to the genera *Candida*, *Meyerozyma*, and *Metschnikowia*, in agreement with other studies [29,84]. The protease activity was strongest in our strains of *H. uvarum*, *L. thermotolerans*, *M. pulcherrima*, *Pichia* spp., *T. delbrueckii*, and *S. cerevisiae* and these results were in agreement with other studies [27,28,32,65,85,86,87] while they were in conflict with Comitini et al. [29] who reported no strains of *L. thermotolerans*, *M. pulcherrima*, *T. delbrueckii*, and *S. cerevisiae* exhibiting any protease activity. Similar negative protease *S. cerevisiae* strains were reported by Charoenchai et al. [33]. Some authors reported no yeasts (including *Candida*, *Debaryomyces*, *Hanseniaspora*, *Metschnikowia*, *Pichia*, *Saccharomyces*, *Torulaspora*) possessing pectinolytic activity [33,77]. Indeed, pectinolytic activity is rarely found in wine-related yeasts but it is reported in *Candida*, *M. pulcherrima*, *L. thermotolerans*, *T. delbrueckii*, *H. uvarum*, and *S. cerevisiae* [21,27,82,88] and our results were in agreement with these authors.

Fermentation vigor is a good indicator of the strain’s promptness and of the progress of the fermentation. It is easy to monitor as a weight measurement, which is directly proportional to sugar consumption and ethanol synthesis, determining the fermentation power. As expected, the *S. cerevisiae* strains had higher fermentation vigor than the non-*Saccharomyces* strains, in agreement with Caridi et al. 2002 [20], with the exception of the *M. pulcherrima* 125/14 strain. This strain, in fact, had higher fermentation vigor than the usually reported 4.5% ethanol (*v*/*v*) [89]. However, it is reported that some strains of *M. pulcherrrima* can produce 9–11.5% ethanol [90], with an ethanol tolerance of at least 6% with a few exceptions above 9% [28].

Acetic acid is one of the compounds that impact the sensory profile of wine, contributing to definitions of its quality. An acetic acid concentration of 0.7–1.1 g/L is considered unpleasant; the maximum acceptable limit for volatile acidity in most wines is 1.2 g/L of acetic acid [91,92]. Values in the range 0.2–0.7 g/L are usually considered optimal [91]. Although most non-*Saccharomyces* are considered high acetic acid producers [93,94] other evidence indicates *T. delbrueckii*, *L. thermotolerans*, *M. pulcherrima* as low producers [29,95,96,97,98,99]. Our results were consistent with these reports, with all selected being within the optimal range with the exception of *H. uvarum* 26/17, the highest producer as reported for the species by Aponte and Blaiotta [99]. However, the behavior of this strain confirmed the utility of the visible halo as a screening test [16,77,100].

The quality of wine is also linked to the glycerol concentration, although this has recently been contested [101]. Noble and Bursick [102] indicated 5.2 g/L as the taste threshold with a maximum acceptable level of 25 g/L [103]. It is usually reported that glycerol production is higher in wines fermented with non-*Saccharomyces* compared to those produced with *S. cerevisiae* [101]. In addition, Zhu et al. [104] reported higher glycerol concentration for non-*Saccharomyces* than for *S. cerevisiae,* which was in agreement with the results of the majority of our tested strains. The role of this metabolite must be considered taking into account its relationship with ethanol and acetic acid production. In fact, higher glycerol production could result in a reduction in ethanol production [105] and a higher production of acetic acid [106].

The role of phenolics in wine is related to the sensorial and health aspects. The effect of SO_2_ in vinification is well known [107] as is the contribution of *S. cerevisiae* to the polyphenolic profile of wine [108,109,110,111,112,113]. Recently, Morata et al. [114] reported different antocyanin adsorption by non-*Saccharomyces* with the goal of improving the color stability of wine. The different polyphenols concentrations in wines produced by our tested yeasts could be attributable to the strain (production of metabolites and cell wall adsorption) used in wines produced without SO_2_ and to the concurrent role of strain and SO_2_ in wines produced with the addition of SO_2_.

Analysis of volatile compounds using GC-MS aimed to determine the production of known and described aroma-active compounds by individual yeast strains. Although the aroma character of some of the compounds has been described as “pleasant“ and their presence in wine is often appreciated, those described as “unpleasant” (or off-flavors) may be very important to achieve the required complexity, fullness and/or typicality of a wine aroma, when present at appropriate concentrations and in certain combinations. Lists of common aroma-active compounds in various types of wine are available in the literature, which facilitates their tracing in experimental samples. However, interpretation of the analytical data need not be straightforward and usually requires combination with sensorial evaluation of the wine bouquet [58,59].

Based on their performance, we propose *S. cerevisiae* PDA W 10, *L. thermotolerans* 5-1-1 and *M. pulcherrima* 125/14 as potential wine starters. Due to the characteristics of *L. thermotolerans* and *M. pulcherrima*, these yeasts are normally used in mixed or sequential fermentations together with *S. cerevisiae* [66,114] in order to complete the fermentation process, guaranteeing the quality of the wine. *S. cerevisiae* PDA W 10 could be used as pure inoculum. Moreover, after further studies on competitive abilities of the strains and examination of the killer trait of *S. cerevisiae* strains, *L. thermotolerans* 5-1-1, *M. pulcherrima* 125/14, and *S. cerevisiae* PDA W 10 could be used as co- or sequential inocula. It must be highlighted that the *M. pulcherrima* 125/14 strain has interesting properties, such as its fermentation vigor, that allows for the consideration of the use of this strain as pure inoculum as well.

The step-by-step process for screening and selecting wine yeasts is as follows: yeast isolation/revitalization of stored yeasts, yeasts identification, Petri plate screening for useful enological characteristics, evaluation of results and choice of strains, use of the chosen strains for micro-vinification, analyses of the produced wines, evaluation of results and identification of wine starter strains.

## 5. Conclusions

Even in the present era of modern molecular technologies, we believe it is important to maintain the know-how of classical methods for selecting wine strains useful for production of high-quality wine. The screening and testing procedures led to the selection of strains that could be the starting point for improvement by hybridization, mutagenesis, or genome engineering. Finally, we believe that this contribution reporting procedures and images could help other scientific groups in screening their own isolated yeasts for wine production.

## Figures and Tables

**Table 1:** Yeast strains used in this study.

Strains	Geographic Origin and Year of Isolation
3-16-1	Bratislava, Slovakia (1958)
5-1-1	Bratislava, Slovakia (1958)
21-1-5	Svätý Jur, Slovakia (1958)
21-1-10	Pezinok, Slovakia (1958)
9-2-1	Bratislava, Slovakia (1959)
12-4-4	Vištuk, Slovakia (1959)
12-4-5	Doľany, Slovakia (1959)
3-16-2	Bratislava, Slovakia (1963)
5-1-3	Bratislava, Slovakia (1965)
11-1-4	Bratislava, Slovakia (1965)
11-1-5	Bratislava, Slovakia (1965)
12-5-1	Bratislava, Slovakia (1965)
5-1-6	Bratislava, Slovakia (1976)
11-1-7	Nitra, Slovakia (1978)
24-1-13	Pezinok, Slovakia (1979)
24-1-25	Mikulov, Czech Republic (1979)
15-1-552	Bratislava, Slovakia (2001)
53	Modra, Slovakia (2009)
125/14	Doľany, Slovakia (2014)
67/14	Doľany, Slovakia (2014)
PDA M 1/1	Modra, Slovakia (2015)
PDA W 9	Modra, Slovakia (2015)
PDA W 10	Modra, Slovakia (2015)
PDA W 11	Modra, Slovakia (2015)
60/16	Strekov, Slovakia (2016)
26/17	Strekov, Slovakia (2017)
CCY 29-178-1	Institute of Chemistry, Slovak Academy of Science, Bratislava, Slovakia
CCY 69-2-15	
CCY 29-97-12	

**Table 2:** Yeast identification by PCR-RFLP analysis of the 5.8-ITS rRNA and sequencing.

Strains	ITS ^a^	RFLP ^b^			Species	% Similarity (Accession No. of the Closest Relative by Blast)
		*Hae*III	*Hinf*I	*Hha*I		
CCY 29-178-1	600	100+500	280+300	280+300	*Candida dubliniensis*	100%/100%-MH545916
5-1-6	750	430	320	310	*Debaryomyces hansenii*	100%/100%-MT183071
9-2-1	775	775	160+200+390	100+320	*Hanseniaspora uvarum*	100%/100%-MN378470
26/17	775	775	160+200+390	100+320		
67/14	800	800	/	310	*Hanseniaspora uvarum*	100%/100%-MN378470
5-1-3	700	210+310	360	300+320	*Lachancea thermotolerans*	100%/100% MK352016
5-1-1	700	100+210+310	330	320	*Lachancea thermotolerans*	100%/100%-MK352016
11-1-4	380	300+410	200	100+220	*Metschnikowia* aff. *chrysoperlae*	100%/100%-JX188172
125/14	400	100+290+400	200	100+210	*Metschnikowia pulcherrima*	100%/96.77%-MK267585
PDA W 11	380	100+290+400	200	100+210		
CCY 69-2-15	400	100+290+400	200	100+210		
11-1-5	380	300	200	100+220	*Metschnikowia pulchrerrima*	100%/99.64%-MN915122
11-1-7	400	100+300	200	100+220	*Metschnikowia pulcherrima*	100%/99.62%-MN915122
12-5-1	750	90+110+420	300+320	280+310	*Meyerozyma guilliermondii*	100%/100%-LC422347
12-4-4	450	90+350	200+290	100+190	*Pichia fermentans*	100%/99.71%-MT645416
12-4-5	450	90+350	200+290	100+190		
CCY 29-97-12	450	90+350	200+290	100+190		
PDA W 9	450	400	210+270	100+180	*Pichia kluyveri*	100%/100%-MN371880
60/16	880	120+180+210+310	110+380	110+370+400	*Saccharomyces cerevisiae*	100%/100%-MT322849
15-1-552	880	120+180+220+310	110+380	110+370+400	*Saccharomyces cerevisiae*	100%/100%-MT641207
53	880	120+180+220+310	110+380	110+370+400		
PDA W 10	880	120+180+220+310	110+380	110+370+400		
PDA M 1/1	880	120+180+220+310	110+380	110+370+400		
3-16-1	800	800	400+450	100+150+220+350	*Torulaspora delbrueckii*	100%/99.67%-MT645452
3-16-2	800	800	400+450	100+150+220+350		
21-1-5	800	800	400+450	100+150+220+350	*Torulaspora delbrueckii*	100%/100%-MT645452
21-1-10	800	800	400+450	100+150+220+350		
24-1-13	800	800	400+450	100+150+220		
24-1-25	800	800	180+210+320	100+290+310	*Zygosaccharomyces bailii*	100%/100%-MF189725

^a^ ITS: Internal Transcribed Spacer. ^b^ Restriction Fragment Length Polymorphism.

**Table 3:** Colony characteristics of yeast strains grown on YPD agar.

Strains	Colony Morphology, Color, and Texture
*Candida dubliniensis* CCY 29-178-1	Flat, cream-colored, wrinkled
*Debaryomyces hansenii* 5-1-6	Convex, white, smooth/glossy
*Hanseniaspora uvarum* 9-2-1	Raised, cream-colored, smooth/glossy
*Hanseniaspora uvarum* 67/14	Raised, cream-colored, smooth/glossy
*Hanseniaspora uvarum* 26/17	Raised, cream-colored, smooth/glossy
*Lachancea thermotolerans* 5-1-3	Umbonate, cream-colored, smooth/glossy
*Lachancea thermotolerans* 5-1-1	Umbonate, cream-colored, smooth/opaque
*Metschnikowia* aff. *chrysoperlae* 11-1-4	Umbonate, cream-colored and red, smooth/opaque
*Metschnikowia pulcherrima* 125/14	Pulvinate, cream-colored and red, smooth/opaque
*Metschnikowia pulcherrima* PDA W 11	Umbonate, cream-colored and red, smooth/opaque
*Metschnikowia pulchrerrima* 11-1-5	Convex, cream-colored and red, smooth/opaque
*Metschnikowia pulcherrima* 11-1-7	Umbonate, cream-colored and red, smooth/opaque
*Metschnikowia pulcherrima* CCY 69-2-15	Convex, cream-colored, smooth/opaque
*Meyerozyma guilliermondii* 12-5-1	Umbonate, cream-colored, smooth/opaque
*Pichia fermentans* 12-4-4	Convex, cream-colored, wrinkled
*Pichia fermentans* 12-4-5	Convex, cream-colored, wrinkled
*Pichia fermentans* CCY 29-97-12	Convex, cream-colored, wrinkled
*Pichia kluyveri* PDA W 9	Umbonate, cream-colored, wrinkled
*Saccharomyces cerevisiae* 60/16	Umbonate, cream-colored, smooth/opaque
*Saccharomyces cerevisiae* 15-1-552	Umbonate, cream-colored, smooth/opaque
*Saccharomyces cerevisiae* 53	Umbonate, cream-colored, smooth/opaque
*Saccharomyces cerevisiae* PDA W 10	Umbonate, cream-colored, smooth/opaque
*Saccharomyces cerevisiae* PDA M 1/1	Umbonate, cream-colored, smooth/opaque
*Torulaspora delbrueckii* 3-16-1	Convex, cream-colored, smooth/opaque
*Torulaspora delbrueckii* 3-16-2	Convex, cream-colored, smooth/opaque
*Torulaspora delbrueckii* 21-1-5	Umbonate, cream-colored, smooth/opaque
*Torulaspora delbrueckii* 21-1-10	Umbonate, cream-colored, smooth/opaque
*Torulaspora delbrueckii* 24-1-13	Umbonate, white, smooth/opaque
*Zygosaccharomyces bailii* 24-1-25	Convex, cream-colored, smooth/opaque

**Table 4:** In vitro tests carried out on the twenty-nine yeast strains.

Strains	Acetic Acid Production ^a^	H_2_S Production ^b^	Ethanol Tolerance ^c^				SO_2_ Resistance ^d^				Catalase ^e^	ß-Glucosidase Activity ^f^	Pectinase Activity ^g^	Esterase Activity ^h^	Protease Activity ^i^
			5%	10%	12%	15%	100 mg/L	200 mg/L	300 mg/L	400 mg/L					
*Candida dubliniensis* CCY 29-178-1	−	dark hazel	+++	+	−	−	*+*	*−*	*−*	*−*	+	−	0.8/++	++	−
*Debaryomyces hansenii* 5−1-6	−	pale hazel	+++	<+	−	−	*−*	*−*	*−*	*−*	+++	+	0.4/−	−	−
*Hanseniaspora uvarum* 9-2-1	+	dark hazel	+++	<+	−	−	*−*	*−*	*−*	*−*	++	−	0.4/−	−	+
*Hanseniaspora uvarum* 67/14	++	dark hazel	+++	<+	−	−	*−*	*−*	*−*	*−*	+++	−	0.4/−	−	+++
*Hanseniaspora uvarum* 26/17	+	dark hazel	+++	<+	−	−	−	*−*	*−*	*−*	++	−	0.4/−	−	++
*Lachancea thermotolerans* 5-1-3	−	hazel	+++	+++	+	<+	*−*	*−*	*−*	*−*	++	−	0.4/+	−	−
*Lachancea thermotolerans* 5-1-1	−	hazel	+++	+	+	<+	*−*	*−*	*−*	*−*	+	−	0.4/−	−	+
*Metschnikowia* aff. *chrysoperlae* 11-1-4	−	hazel	++	<+	−	−	+	+	*−*	*−*	+	++++	0.4/+	++	−
*Metschnikowia pulcherrima* 125/14	−	hazel	+++	+	+	<+	*++*	*+*	*−*	*−*	++	+++	0.4/−	+	−
*Metschnikowia pulcherrima* PDA W 11	−	dark hazel	+++	+	−	−	+	−	−	−	+++	++++	0.5/−	−	++
*Metschnikowia pulchrerrima* 11-1-5	−	hazel	+++	+	−	−	+	−	*−*	*−*	+	++	0.4/+	++	+++
*Metschnikowia pulcherrima* 11-1-7	−	hazel	+++	+	−	−	*++*	*−*	*−*	*−*	++	++++	0.4/+	−	+++
*Metschnikowia pulcherrima* CCY 69-2-15	−	hazel	+++	<+	−	−	++	+	−	*−*	+++	++++	0.4/+	−	−
*Meyerozyma guilliermondii* 12-5-1	−	dark hazel	++	+	−	−	*−*	*−*	*−*	*−*	+	+++	0.4/−	+	−
*Pichia fermentans* 12-4-4	−	black	+++	+	−	−	*+*	*−*	*−*	*−*	++	−	0.6/−	−	+++
*Pichia fermentans* 12-4-5	−	black	+++	+	−	−	++	*−*	*−*	*−*	++	−	0.5/−	−	++
*Pichia fermentans* CCY 29-97-12	+	black	+++	+	−	−	+	*−*	*−*	*−*	+++	−	0.6/−	−	+
*Pichia kluyveri* PDA W 9	−	black	+++	+++	+	−	+++	++	+	*−*	++	++++	0.4/++	−	++
*Saccharomyces cerevisiae* 60/16	−	hazel	+++	++	+	+	*+++*	*++*	*−*	*−*	+	−	0.4/<+	−	+
*Saccharomyces cerevisiae* 15-1-552	−	hazel	+++	++	+	+	*+++*	*+++*	*+++*	*+++*	+	−	0.4/<+	−	+
*Saccharomyces cerevisiae* 53	−	hazel	+++	++	+	<+	*+++*	*+++*	*+++*	*+++*	+	−	0.4/<+	−	++
*Saccharomyces cerevisiae* PDA W 10	−	hazel	+++	++	+	+	*+++*	*+++*	*+++*	*+++*	+	−	0.4/+	−	+
*Saccharomyces cerevisiae* PDA M 1/1	−	hazel	+++	+++	++	++	*+++*	*+++*	*+++*	*+++*	+	−	0.4/−	−	+
*Torulaspora delbrueckii* 3-16-1	−	dark hazel	+++	+	<+	<+	*++*	*+*	*−*	*−*	+++	−	0.4/+	−	−
*Torulaspora delbrueckii* 3-16-2	−	dark hazel	+++	+	<+	<+	++	*+*	*−*	*−*	++	−	0.4/+	−	−
*Torulaspora delbrueckii* 21-1-5	−	dark hazel	+++	+	+	<+	*+*	*−*	*−*	*−*	+	−	0.4/−	−	−
*Torulaspora delbrueckii* 21-1-10	−	dark hazel	+++	+	−	−	*+*	−	−	*−*	+	−	0.4/−	−	+
*Torulaspora delbrueckii* 24-1-13	−	dark hazel	+++	+	−	−	*+*	−	−	*−*	+++	−	0.4/−	−	−
*Zygosaccharomyces bailii* 24-1-25	−	white	+++	+	+	−	*+*	*+*	*+*	*+*	++	−	0.4/−	−	−

^a^ Halo: −, none; +, low; ++, medium. ^b^ Biomass color. ^c^ Growth observed after 1 d of incubation: −, no growth; <+, very weak; +, weak; ++, good; +++, optimal. ^d^ Added as potassium metabisulphite. Growth observed after 1 d of incubation: −, no; +,weak; ++, good; +++, optimal. ^e^ Development of bubbles: +, low; ++, medium, +++, high. ^f^ Color of the medium from none (−) to dark color (++++). ^g^ Mean of colony diameter in mm/halo. −, no halo; <+, very faint halo; +, faint halo; ++, clear halo. ^h^ Activity: −, no opaque halo; +, faint opaque halo; ++, strong opaque halo. ^i^ Activity: −, no halo; +, small diameter; ++, medium diameter; +++, large diameter.

**Table 5:** Mean values of fermentation vigor, expressed as g CO_2_/100 mL, of the eleven yeast strains tested in red grape must.

Strains	Fermentation Vigor	after 2 d	Fermentation Vigor	after 7 d
	w/o SO_2_	with SO_2_	w/o SO_2_	with SO_2_
*Debaryomyces hansenii* 5-1-6	0.7 ± 0.07 ^g^	0.6 ± 0.10 ^e^	2.2 ± 0.06 ^h^	2.1 ± 0.04 ^l^
*Hanseniaspora uvarum* 26/17	1.4 ± 0.06 ^f^	1.3 ± 0.18 ^d^	5.3 ± 0.04 ^f^	5.5 ± 0.13 ^h^
*Lachancea thermotolerans* 5-1-1	2.1 ± 0.20 ^e^	2.8 ± 0.03 ^b^	8.1 ± 0.21 ^d^	9.7 ± 0.17 ^e^
*Metschnikowia pulcherrima* 125/14	2.5 ± 0.06 ^d^	3.0 ± 0.25 ^ab^	10.5 ± 0.13 ^b^	10.8 ± 0.14 ^b^
*Metschnikowia pulcherrima* 11-1-7	0.8 ± 0.03 ^g^	0.8 ± 0.11 ^e^	3.1 ± 0.07 ^g^	3.0 ± 0.01 ^i^
*Saccharomyces cerevisiae* 15-1-552	3.5 ± 0.14 ^b^	3.2 ± 0.07 ^a^	10.6 ± 0.04 ^b^	11.2 ± 0.21 ^a^
*Saccharomyces cerevisiae* 53	2.9 ± 0.07 ^c^	3.0 ± 0.21 ^ab^	10.2 ± 0.11 ^c^	10.7 ± 0.04 ^b^
*Saccharomyces cerevisiae* PDA W 10	3.8 ± 0.06 ^a^	3.2 ± 0.02 ^a^	10.9 ± 0.02 ^a^	10.0 ± 0.01 ^d^
*Saccharomyces cerevisiae* PDA M 1/1	2.9 ± 0.28 ^c^	3.3 ± 0.14 ^a^	10.0 ± 0.08 ^c^	10.4 ± 0.11 ^c^
*Torulaspora delbrueckii* 3-16-1	1.6 ± 0.03 ^f^	1.7 ± 0.04 ^c^	7.6 ± 0.01 ^e^	7.2 ± 0.07 ^f^
*Zygosaccharomyces bailii* 24-1-25	0.9 ± 0.03 ^g^	1.2 ± 0.01 ^d^	5.4 ± 0.25 ^f^	5.9 ± 0.03 ^g^
None (un-inoculated must)	0.2 ± 0.01 ^h^	0.1 ± 0.02 ^f^	0.2 ± 0.01 ^i^	0.2 ± 0.01 ^m^

Values in a column with identical superscript letters are not statistically different according to one-way ANOVA with Tukey’s test at a statistical significance level of 0.05.

**Table 6:** pH, total titratable acidity, and volatile acidity of the wines produced by inoculating the red must without and with SO_2_ for the eleven yeast strains.

Strains	pH		Total Titratable Acidity		Volatile Acidity	
	w/o SO_2_	with SO_2_	w/o SO_2_	with SO_2_	w/o SO_2_	with SO_2_
*Debaryomyces hansenii* 5-1-6	3.47 ± 0.03 ^b^	3.34 ± 0.02 ^a^	5.15 ± 0.01	5.22 ±0.03	0.12 ± 0.00 ^a^	0.48 ± 0.01 ^b^
*Hanseniaspora uvarum* 26/17	3.34 ± 0.02 ^a^	3.29 ± 0.01	8.24 ± 0.01	7.95 ± 0.02	1.68 ± 0.01	1.56 ± 0.01
*Lachancea thermotolerans* 5-1-1	3.44 ± 0.03 ^ab^	3.41 ± 0.01 ^ab^	6.55 ± 0.04 ^c^	6.84 ± 0.01 ^b^	0.12 ± 0.01 ^a^	0.14 ± 0.01 ^a^
*Metschnikowia pulcherrima* 125/14	3.43 ± 0.01 ^ab^	3.36 ± 0.03 ^a^	6.92 ± 0.03 ^a^	6.84 ± 0.01 ^b^	0.48 ± 0.03 ^b^	0.18 ± 0.01
*Metschnikowia pulcherrima* 11-1-7	3.34 ± 0.02 ^a^	3.33 ± 0.01 ^a^	5.70 ± 0.14 ^b^	5.96 ± 0.00	0.12 ± 0.01 ^a^	0.06 ± 0.01
*Saccharomyces cerevisiae* 15-1-552	3.46 ± 0.06 ^b^	3.44 ± 0.02 ^b^	6.48 ± 0.01 ^c^	6.29 ± 0.01	0.36 ± 0.00	0.48 ± 0.00 ^b^
*Saccharomyces cerevisiae* 53	3.48 ± 0.04 ^b^	3.40 ± 0.02 ^ab^	6.84 ± 0.02 ^a^	6.84 ± 0.01 ^b^	0.12 ± 0.00 ^a^	0.48 ± 0.00 ^b^
*Saccharomyces cerevisiae* PDA W 10	3.44 ± 0.01 ^ab^	3.41 ± 0.01 ^ab^	6.48 ± 0.00 ^c^	6.22 ± 0.01	0.48 ± 0.02 ^b^	0.12 ± 0.01 ^a^
*Saccharomyces cerevisiae* PDA M 1/1	3.48 ± 0.04 ^b^	3.36 ±0.01 ^a^	6.55 ± 0.00 ^c^	6.59 ± 0.00 ^a^	0.48 ± 0.01 ^b^	0.48 ±0.02 ^b^
*Torulaspora delbrueckii* 3-16-1	3.42 ± 0.02 ^ab^	3.37 ± 0.01 ^a^	6.95 ± 0.06 ^a^	6.55 ± 0.01 ^a^	0.09 ± 0.01 ^a^	0.12 ± 0.00 ^a^
*Zygosaccharomyces bailii* 24-1-25	3.41 ± 0.01 ^ab^	3.41 ± 0.01 ^ab^	6.40 ± 0.01 ^c^	6.11 ± 0.01	0.24 ± 0.02	0.30 ± 0.01
None (un-inoculated must)	3.22 ± 0.01	3.26 ± 0.00	5.67 ± 0.01 ^b^	5.15 ± 0.02	n.d.	n.d.

Values represent mean ± Standard Deviation (SD) from two measurements. Values in a column with identical superscript letters are not statistically different according to one-way ANOVA with Tukey’s test at a statistical significance level of 0.05. n.d.: not detected.

**Table 7:** Ethanol, glucose, and fructose content of the wines produced by inoculating the red must without and with SO_2_ for the eleven yeast strains.

Strains	Ethanol (%, *v*/*v*)		Glucose (g/L)		Fructose (g/L)	
	w/o SO_2_	with SO_2_	w/o SO_2_	with SO_2_	w/o SO_2_	with SO_2_
*Debaryomyces hansenii* 5-1-6	1.00 ± 0.00	0.80 ± 0.02	75.73 ± 1.26	76.88 ± 0.29	75.02 ± 0.21	76.22 ± 0.25
*Hanseniaspora uvarum* 26/17	5.55 ± 0.01	5.41 ± 0.01 ^b^	43.87 ± 0.53	44.65 ± 0.33	39.71 ± 0.24	38.55 ± 0.04
*Lachancea thermotolerans* 5-1-1	7.25 ± 0.01 ^a^	7.15 ± 0.00 ^a^	5.28 ± 0.07 ^a^	<LOD	14.21 ± 0.14 ^a^	1.39 ± 0.13
*Metschnikowia pulcherrima* 125/14	7.10 ± 0.03 ^a^	6.45 ± 0.04 ^a^	<LOD	<LOD	0.22 ± 0.04 ^b^	0.18 ± 0.06
*Metschnikowia pulcherrima* 11-1-7	4.15 ± 0.07 ^b^	4.10 ± 0.07 ^b^	52.61 ± 1.97	55.47 ± 1.94	60.60 ± 0.40	63.25 ± 2.84
*Saccharomyces cerevisiae* 15-1-552	8.39 ± 0.03 ^c^	7.34 ± 0.03 ^a^	~LOD	<LOD	<LOD	<LOD
*Saccharomyces cerevisiae* 53	8.35 ± 0.01 ^c^	8.11 ± 0.01 ^a^	<LOD	<LOD	<LOD	<LOD
*Saccharomyces cerevisiae* PDA W 10	8.15 ± 0.07	8.23 ± 0.00 ^a^	<LOD	<LOD	<LOD	0.08 ± 0.01
*Saccharomyces cerevisiae* PDA M 1/1	7.15 ± 0.07 ^a^	7.43 ± 0.03 ^a^	<LOD	<LOD	<LOD	<LOD
*Torulaspora delbrueckii* 3-16-1	7.13 ± 0.04 ^a^	7.11 ± 0.00 ^a^	4.60 ± 0.37 ^a^	5.00 ± 1.41	13.65 ± 1.01 ^a^	14.72 ± 0.65
*Zygosaccharomyces bailii* 24-1-25	6.71 ± 0.00	4.84 ± 2.09 ^b^	56.27 ± 2.09	49.40 ± 0.40	1.06 ± 0.23 ^b^	<LOD
None (un-inoculated must)	n.d.	n.d.	85.38 ± 0.14	85.11 ± 1.69	83.98 ± 1.15	82.85 ± 0.52

Values represent mean ± SD from three measurements. Values in a column with identical superscript letters are not statistically different according to one-way ANOVA with Tukey’s test at a statistical significance level of 0.05. n.d.: not detected. Analytical parameters for glucose determination were: limit of detection (LOD) = 0.08 g/L, limit of quantification (LOQ) = 0.10 g/L. Analytical parameters for fructose determination were: LOD = 0.04 g/L, LOQ = 0.06 g/L.

**Table 8:** Glycerol, total polyphenol, and total flavonoids content of the wines produced by inoculating the red must without and with SO_2_ for the eleven yeast strains.

Strains	Glycerol (g/L)		Total Polyphenols (GAE, mg/L)		Total Flavonoids (QE, mg/L)	
	w/o SO_2_	with SO_2_	w/o SO_2_	with SO_2_	w/o SO_2_	with SO_2_
*Debaryomyces hansenii* 5-1-6	0.50 ± 0.04 ^d^	0.31 ± 0.04	895.23 ± 6.17	940.45 ± 7.55 ^d^	59.49 ± 1.04 ^b^	68.90 ± 0.86 ^bd^
*Hanseniaspora uvarum* 26/17	3.63 ± 0.21 ^bc^	3.88 ± 0.00 ^c^	921.85 ± 5.42 ^b^	938.52 ± 5.16 ^bd^	50.74 ± 0.62 ^a^	58.78 ± 1.15 ^a^
*Lachancea thermotolerans* 5-1-1	4.43 ± 0.01 ^a^	5.17 ± 0.13 ^d^	779.30 ± 1.72	753.00 ± 7.56	51.88 ± 0.13 ^a^	51.89 ± 0.72
*Metschnikowia pulcherrima* 125/14	4.19 ± 0.04 ^a^	4.30 ± 0.01 ^b^	920.11 ± 0.40 ^b^	918.83 ± 0.32 ^b^	59.10 ± 0.87 ^b^	70.20 ± 1.27 ^b^
*Metschnikowia pulcherrima* 11-1-7	6.31 ± 0.30	6.53 ± 0.04	867.45 ± 6.01 ^a^	987.01 ± 6.95 ^c^	52.77 ± 0.43 ^a^	61.05 ± 1.48 ^a^
*Saccharomyces cerevisiae* 15-1-552	3.85 ± 0.25 ^ab^	3.86 ± 0.02 ^ac^	905.87 ± 14.13 ^b^	948.42 ± 6.05 ^d^	67.31 ± 0.32 ^c^	76.48 ± 2.62 ^c^
*Saccharomyces cerevisiae* 53	3.37 ± 0.18 ^c^	3.62 ± 0.00	905.26 ± 0.86 ^b^	958.43 ± 2.15 ^d^	63.10 ± 0.01 ^d^	69.55 ± 1.29 ^bd^
*Saccharomyces cerevisiae* PDA W 10	4.46 ± 0.04 ^a^	4.31 ± 0.14 ^b^	982.85 ± 12.05	925.86 ± 2.65 ^b^	69.44 ± 1.03 ^c^	67.93 ± 1.03 ^d^
*Saccharomyces cerevisiae* PDA M 1/1	3.65 ± 0.05 ^bc^	4.17 ± 0.06 ^b^	944.61 ± 13.01 ^b^	979.51 ± 3.61 ^c^	70.83 ± 0.28 ^c^	77.55 ± 1.54 ^c^
*Torulaspora delbrueckii* 3-16-1	4.24 ± 0.25 ^a^	3.66 ± 0.13 ^a^	854.27 ± 6.86 ^a^	836.21 ± 5.33 ^a^	51.04 ± 0.49 ^a^	57.36 ± 1.66 ^a^
*Zygosaccharomyces bailii* 24-1-25	5.42 ± 0.06	5.35 ± 0.05 ^d^	968.26 ± 7.45	935.99 ± 2.54 ^b^	65.89 ± 0.72 ^c^	65.65 ± 0.41 ^d^
None (un-inoculated must)	0.23 ± 0.04 ^d^	<LOD	914.79 ± 2.55 ^b^	994.98 ± 0.65 ^c^	61.55 ± 0.97 ^d^	80.28 ± 0.49 ^c^

Values represent mean ± SD from three measurements. Values in a column with identical superscript letters are not statistically different according to one-way ANOVA with Tukey’s test at a statistical significance level of 0.05. Analytical parameters for glycerol determination were: LOD = 0.22 g/L, LOQ = 0.40 g/L.

## Data Availability

The data presented in this study are available in Sidari, R.; Ženišová, K.; Tobolková, B.; Belajová, E.; Cabicarová, T.; Bučková, M.; Puškárová, A.; Planý, M.; Kuchta, T.; Pangallo, D.; Wine yeasts selection: an experimental review by images; Microorganisms and its supplementary material.

## Supplementary Materials

The following are available online at https://www.mdpi.com/article/10.3390/microorganisms9112223/s1: Table S1. Volatile organic compounds of wines with SO_2_ produced using the eleven yeast strains, Table S2. Volatile organic compounds of wines without SO_2_ produced using the eleven yeast strains.
