# Supplementary material for: Wine Yeasts Selection: Laboratory Characterization and Protocol Review
Source: Microorganisms. 2021 Oct 26;9(11):2223. doi: 10.3390/microorganisms9112223 (PMC8623447; doi:10.3390/microorganisms9112223)
Supplement: Supplementary file 1 [file microorganisms-09-02223-s001.zip › microorganisms-1365579-supplementary.pdf]

Supplementary Materials

Table S1. Volatile organic compounds of the wines with SO_2 produced using the eleven yeast strains.

Compound	Peak area %*											
	T. delbrueckii 3-16-1	*M. bicuspidata* 125/14	*M. pulcherrima* 11-1-7	*S. cerevisiae* 15-1-552	*S. cerevisiae* 53	*S. cerevisiae* PDA W 10	*S. cerevisiae* PDA M 1/1	*D. hansenii* 5-1-6	*L. thermotolerans* 5-1-1	*Z. bailii* 24-1-25	*H. uvarum* 26/17	Un-inoculated
Acetaldehyde	0.84	0.76	0.00	1.29	2.03	1.16	0.99	0.00	0.33	0.54	0.24	0.00
1-Propanol	0.00	0.00	0.00	0.00	0.00	0.00	0.00	0.00	0.00	0.63	0.00	0.00
Isobutanol	0.00	0.00	0.23	0.00	0.00	0.00	0.00	0.00	0.00	0.00	0.00	0.00
3-Methyl-1-butanol	6.21	7.14	5.33	7.86	12.29	14.60	11.38	1.01	12.27	4.23	1.28	0.00
Ethyl hexanoate	0.00	0.48	0.00	0.36	0.00	0.94	0.90	0.00	0.36	0.00	0.00	0.00
Hydroxyacetone	0.18	0.00	0.36	0.00	0.00	0.00	0.00	0.61	0.16	0.49	0.37	0.50
Acetoin	0.00	0.00	0.00	0.00	0.00	0.00	0.00	0.00	0.00	0.58	1.44	0.00
1-Hexanol	0.00	0.00	0.20	0.00	0.00	0.00	0.00	0.68	0.00	0.00	0.00	0.42
Ethyl octanoate	0.00	0.46	0.00	1.03	0.00	2.10	1.25	0.00	0.00	0.00	0.00	0.00
Acetic acid	3.12	7.05	3.25	7.92	12.02	7.32	9.42	5.85	3.77	5.87	12.01	4.49
Furfural	3.49	0.59	6.26	0.52	0.44	0.00	0.28	8.17	0.98	4.12	6.16	10.15
2-Hydroxyfuraneol	0.36	0.00	0.65	0.00	0.00	0.00	0.00	0.99	0.00	0.71	0.84	0.00
2,3-Butanediol	2.33	2.44	0.00	3.85	5.54	2.23	3.12	0.00	2.86	1.03	0.00	0.00
5-Methylfurfural	0.72	0.38	0.75	0.47	0.48	0.45	0.46	1.03	0.54	0.82	0.89	0.00
Ethyl decanoate	0.00	0.33	0.00	0.36	0.00	0.71	0.47	0.00	0.00	0.00	0.00	0.00
2-Furanmethanol	0.76	0.14	1.25	0.00	0.00	0.00	0.18	2.03	0.48	1.30	1.22	1.53
Ethyl 9-decenoate	0.00	0.00	0.00	0.00	0.00	0.30	0.00	0.00	0.00	0.00	0.00	0.00
Phenylethyl acetate	0.00	0.23	0.00	0.35	0.48	0.40	0.27	0.00	0.00	1.46	0.21	0.00
Benzyl alcohol	0.00	0.00	0.14	0.00	0.00	0.00	0.00	0.38	0.00	0.00	0.00	0.19
2-Phenylethanol	10.32	11.14	7.10	15.36	17.03	17.71	12.31	1.23	22.37	8.42	4.71	0.43
Furyl hydroxymethylketone	0.60	0.00	0.47	0.00	0.00	0.00	0.00	0.95	0.00	0.54	0.81	0.00
Octanoic acid	0.00	1.18	0.00	2.22	0.27	3.81	2.31	0.00	0.30	0.09	0.37	0.00
4-Vinylguaiacol	0.00	0.57	0.00	0.73	0.00	0.84	0.75	1.38	0.00	0.00	0.00	0.00
Decanoic acid	0.32	3.89	0.00	5.69	0.37	7.16	5.67	0.00	0.84	0.00	0.45	0.11
Pyranone	8.41	1.54	9.05	1.55	0.89	0.40	0.60	12.77	4.51	8.79	9.74	14.07
Hydroxymaltol	0.38	0.00	0.60	0.00	0.00	0.00	0.00	0.80	0.00	1.29	1.00	0.93
9-Decenoic acid	0.00	2.13	0.00	2.84	0.00	4.29	3.08	0.00	0.00	0.00	0.00	0.00
Coumaran	0.00	0.90	0.00	1.73	1.64	2.02	1.43	1.59	0.00	0.21	0.19	0.00
Dodecanoic acid	0.00	0.00	0.00	0.69	0.43	0.00	0.44	0.49	0.32	0.00	0.00	0.00
5-Hydroxymethyfurfural	0.00	4.46	3.05	1.29	1.40	0.00	8.80	21.82	30.82	0.00	0.00	4.46
1-(2-Furanyl)-1,2-ethanediol	2.60	3.98	3.81	2.75	4.65	3.18	3.78	4.00	2.14	0.00	2.60	3.98

* Area percent of glycerol was removed as it is not reliably analyzed by the method. From the un-inoculated sample, area percent of 1-ethyl-2-hydroxymethylimidazole (51.48) was also removed.

Table S2. Volatile organic compounds of the wines without SO_2 produced using the eleven yeast strains.

Compound	Peak area %*											
	T. delbrueckii 3-16-1	*M. bicuspidata* 125/14	*M. pulcherrima* 11-1-7	*S. cerevisiae* 15-1-552	*S. cerevisiae* 53	*S. cerevisiae* PDA W 10	*S. cerevisiae* PDA M 1/1	*D. hansenii* 5-1-6	*L. thermotolerans* 5-1-1	*Z. bailii* 24-1-25	*H. uvarum* 26/17	Un-inoculated
Acetaldehyde	0.82	0.67	0.00	0.94	2.21	0.56	1.12	0.00	0.83	0.44	0.28	0.00
1-Propanol	0.40	0.46	0.00	0.60	0.57	0.30	0.00	0.00	0.31	0.58	0.00	0.00
Isobutanol	0.00	0.00	0.18	0.25	0.48	0.00	0.00	0.00	0.00	0.00	0.00	0.00
3-Methyl-1-butanol	8.75	13.10	4.60	12.89	16.31	12.85	10.25	1.54	9.32	2.95	1.63	0.00
Ethyl hexanoate	0.24	3.39	0.00	0.00	2.00	1.65	0.00	0.00	0.09	0.00	0.00	0.00
Hydroxyacetone	0.00	0.00	0.35	0.00	0.00	0.00	0.00	0.58	0.24	0.38	0.00	0.00
Acetoin	0.00	0.00	0.00	0.00	0.85	0.00	0.00	0.00	0.26	0.00	1.43	0.00
1-Hexanol	0.00	0.27	0.18	0.21	0.00	0.26	0.00	1.02	0.30	0.00	0.00	0.00
Ethyl octanoate	0.00	2.21	0.00	0.88	0.42	1.30	0.43	0.00	0.00	0.00	0.00	0.00
Acetic acid	2.00	6.03	3.05	6.99	9.17	5.49	10.31	5.21	3.86	6.54	16.25	3.51
Furfural	3.41	0.40	5.50	0.20	0.00	0.00	0.22	8.63	1.81	4.13	5.21	0.00
2-Hydroxyfuraneol	0.00	0.00	0.57	0.00	0.00	0.00	0.00	0.86	0.41	0.61	0.60	0,00
2,3-Butanediol	2.68	2.19	0.00	3.73	3.75	2.04	3.27	0.00	3.20	0.77	0.00	0.00
5-Methylfurfural	0.72	0.49	0.50	0.35	0.30	0.29	0.31	0.85	0.53	0.69	0.57	0.00
Propylene glycol	0.00	0.28	0.00	0.49	0.00	0.00	0.19	0.00	0.00	0.00	0.00	0.00
4-Cyclopentene-1,3-dione	0.00	0.00	0.17	0.00	0.00	0.00	0.00	0.32	0.00	0.19	0.15	0.00
Diethyleneglycol ethylether	0.00	0.35	0.00	0.34	0.00	0.31	0.00	0.00	0.37	0.00	0.00	0.00
Ethyl decanoate	0.00	1.80	0.00	0.89	0.00	0.75	0.21	0.00	0.00	0.00	0.00	0.00
2-Furanmethanol	0.43	0.27	1.26	0.22	0.00	0.19	0.18	1.99	0.64	0.99	1.00	0.00
Ethyl 9-decenoate	0.00	0.39	0.00	0.18	0.00	0.29	0.00	0.00	0.00	0.00	0.00	0.00
2-Methylthiolane	0.00	0.00	0.00	0.00	0.00	0.00	0.00	0.00	0.25	0.00	0.00	0.00
Phenylethyl acetate	0.00	1.07	0.00	0.73	0.85	0.86	0.28	0.00	0.00	0.84	0.26	0.00
Hexanoic acid	0.00	0.58	0.00	0.38	0.33	0.47	0.22	0.00	0.08	0.00	0.00	0.00
Benzyl alcohol	0.00	0.00	0.00	0.00	0.00	0.00	0.00	0.39	0.00	0.00	0.00	0.15
2-Phenylethanol	13.30	15.90	6.93	13.49	22.90	14.94	11.53	1.77	19.36	8.15	4.71	1.47
Furyl hydroxymethylketone	0.00	0.00	0.00	0.18	0.00	0.00	0.00	0.85	0.38	0.51	0.44	0.00
Octanoic acid	0.26	5.05	0.00	3.44	0.89	4.79	3.31	0.00	0.88	0.00	0.33	0.14
Nonanoic acid	0.00	0.41	0.00	0.16	0.00	0.00	0.00	0.00	0.00	0.00	0.00	0.00
4-Vinylguaiacol	0.00	0.97	0.00	0.73	1.41	0.64	0.77	0.00	0.00	0.00	0.00	0.00
Decanoic acid	1.94	10.97	0.00	6.85	0.98	9.18	7.43	0.00	1.09	0.00	0.41	0.00
Pyranone	3.56	0.66	10.25	0.91	0.34	0.56	0.67	12.14	3.91	7.93	8.23	11.38
Hydroxymaltol	0.35	0.00	0.62	0.00	0.00	0.00	0.00	0.75	0.55	0.51	0.55	0.62
9-Decenoic acid	1.97	3.52	0.00	3.30	0.00	4.08	3.52	0.00	0.00	0.00	0.00	0.00
Coumaran	0.00	1.58	0.00	1.45	2.53	1.21	1.65	1.25	0.00	0.00	0.00	0.00
5-Hydroxymethyfurfural	27.37	1.57	35.79	0.48	0.95	0.57	0.73	48.93	14.95	32.11	34.59	0.00
1-(2-Furanyl)-1,2-ethanediol	0.00	0.00	2.64	2.79	3.38	2.87	4.15	3.27	3.67	3.53	2.83	0.00

* Area percent of glycerol was removed as it is not reliably analyzed by the method. From the un-inoculated sample, area percent of 1-ethyl-2-hydroxymethylimidazole (59.00) was also removed.
